# Location, location, location: environmental factors better predict malaria-positive individuals during reactive case detection than index case demographics in Southern Province, Zambia

**DOI:** 10.1186/s12936-016-1649-z

**Published:** 2017-01-06

**Authors:** David A. Larsen, Tokozile Ngwenya-Kangombe, Sanford Cheelo, Busiku Hamainza, John Miller, Anna Winters, Daniel J. Bridges

**Affiliations:** 1 Department of Public Health, Food Studies and Nutrition, Syracuse University, 344D White Hall, Syracuse, NY 13244 USA; 2 Akros, Lusaka, Zambia; 3 National Malaria Control Centre, Lusaka, Zambia; 4 PATH MACEPA, Lusaka, Zambia; 5 University of Montana School of Public and Community Health Science, Missoula, MT USA

**Keywords:** Surveillance, Elimination, Reactive case detection, Hot spot

## Abstract

**Background:**

Decreasing malaria transmission leads to increasing heterogeneity with increased risk in both hot spots (locations) and hot pops (certain demographics). In Southern Province, Zambia, reactive case detection has formed a part of malaria surveillance and elimination efforts since 2011. Various factors may be associated with finding malaria infections during case investigations, including the demographics of the incident case and environmental characteristics of the location of the incident case.

**Methods:**

Community health worker registries were used to determine what factors were associated with finding a malaria infection during reactive case detection.

**Results:**

Location was a more powerful predictor of finding malaria infections during case investigations than the demographics of the incident case. After accounting for environmental characteristics, no demographics around the incident case were associated with finding malaria infections during case investigations. Various time-invariant measures of the environment, such as median enhanced vegetation index, the topographic position index, the convergence index, and the topographical wetness index, were all associated as expected with increased probability of finding a malaria infection during case investigations.

**Conclusions:**

These results suggest that targeting the locations highly at risk of malaria transmission is of importance in elimination settings.

## Background

As malaria transmission decreases it becomes increasingly heterogeneous, with pockets of residual transmission when approaching elimination [1]. Identifying and clearing these pockets is key for malaria elimination programmes to succeed [2]. Population-based surveys of malaria parasite prevalence have been employed to find clusters of transmission, e.g., in Sudan [3], while school-based surveys have also been trialled in The Gambia [4]. Unfortunately, as transmission drops to low levels the utility of these population-based surveys for identifying residual transmission rapidly declines. Furthermore, these surveys are unwieldy, expensive, have limited spatial resolution or coverage, and are constrained to a single snapshot in time unless repeated.

In contrast, routine malaria surveillance can serve as both a tool for planning and evaluating malaria interventions, as well as a method of targeting malaria interventions to residual foci of malaria transmission in elimination settings [5]. Care needs to be taken with passive case detection data, i.e., derived from self-reported symptomatic individuals. As these data are greatly influenced by treatment-seeking behaviour [6]. Nevertheless, the sensitivity of routine malaria surveillance, i.e., the ability to detect a greater proportion of the reservoir of human infections [7], can be enhanced by extending services into the community and integrating reactive case detection (RCD) for confirmed cases (Larsen et al., pers. comm.). Briefly, RCD consists of performing case investigations at and around the home of an incident malaria case [8, 9]. RCD is based on the assumption that incident malaria cases are indicative of local malaria transmission and therefore an increased risk of infection in individuals living in close proximity to the incident case [10–12]. In Zambia, RCD has been scaled throughout all low transmission areas (incidence of <5 per 1000) and involves testing all individuals within 140 m of an incident malaria case with a rapid diagnostic test (RDT) and treating those positive with artemether–lumefantrine [13].

The probability that an incident malaria case indicates additional malaria infections within the range of an RCD response due to local transmission is likely influenced by a number of factors related to the characteristics and behaviour of both the incident malaria case and the surrounding community members [14]. For the incident case, a key determinant is travel history, i.e., travel from an area of low to high transmission may be indicative of imported malaria. In Zambia travel history is only recorded when the individual has travelled outside of their home district in the previous month. Assuming progression to a symptomatic infection and presentation to a health facility occurs within a month, the absence of travel suggests that transmission has occurred within a district. Within the RCD population, certain demographics are more likely to harbour malaria infections than others, due to their exposure to malaria-transmitting mosquitoes [15]. Characteristics such as gender, age, occupation as well as travel history may all be associated with increased or decreased probability of finding malaria infections during a response. The utility of incident malaria cases as a proxy for detecting pockets of residual transmission is also heavily influenced by local environmental factors that drive vector abundance. Ultimately, heterogeneity in *Anopheles* mosquito habitat drives observed heterogeneity in malaria transmission [15]. For example, during the dry season *Anopheles* mosquito habitat is clustered around more permanent breeding sites [16], whereas habitat expands dramatically during the wet season, suggesting that an incident case in the wet season may be less likely to be associated with localized transmission. Topographical measures may also influence where additional malaria cases are found due to their capacity to predict larval breeding sites [17–20], and have been linked with malaria risk in some instances [21, 22] but not in others [23]. More clearly understanding factors influencing the probability of finding additional malaria positives will help malaria control and elimination programmes to identify residual pockets of malaria transmission and target interventions to them to progress to elimination.

This paper examines how characteristics of incident malaria cases seeking care, as well as environmental factors, are associated with the probability of finding malaria infections during RCD.

## Methods

### Study area

**Fig. 1:**
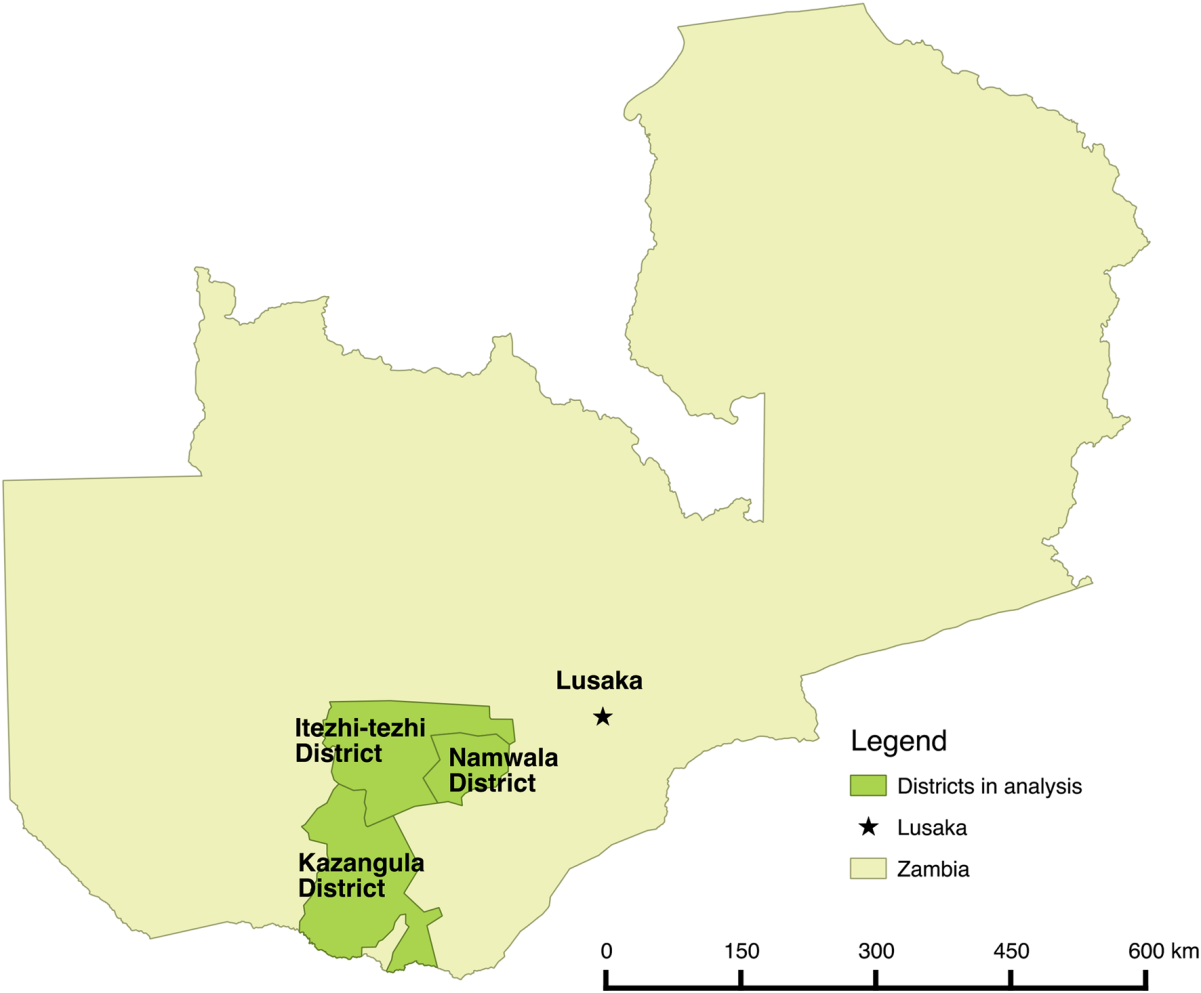
Map of the study area

**Fig. 2:**
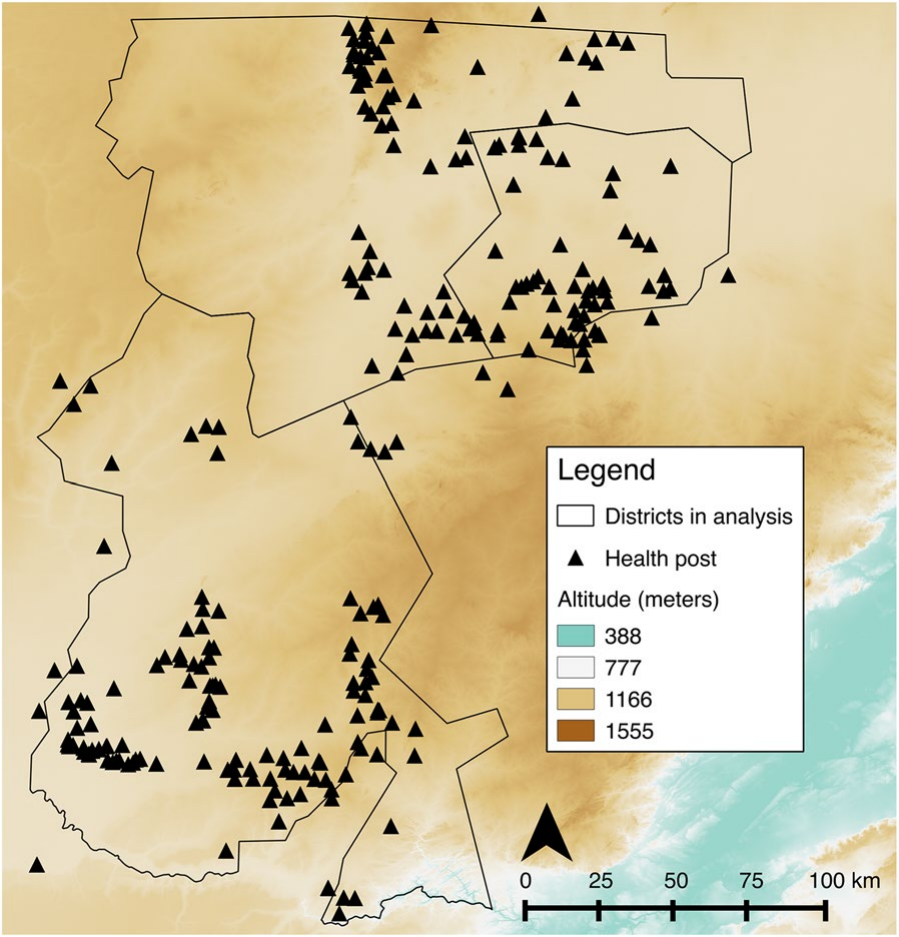
Altitude of the study area

Southern Province, Zambia has low malaria transmission with previous malaria indicator surveys finding <5% malaria parasite prevalence in children <5 years of age. The districts near Lake Kariba have the highest malaria transmission intensity, with transmission intensity waning further inland (north and northwest) from the Lake [24]. Due to such low transmission, Southern Province contains the first districts targeted for elimination in the national strategic plan. This study analysed data from three districts: Itezhi-tezhi, Kazungula and Namwala, in Southern Province (Fig. 1) which are in pre-elimination phase. Subsistence farming is the principal means of employment in the area, and the major malaria vector is *Anopheles arabiensis.* Ecologically the area is a relatively flat plain dominated by scrub forest (Fig. 2).

### Case investigations

RCD has formed part of the Ministry of Health’s routine malaria surveillance in Southern Province, Zambia since 2011 [13]. In brief, volunteer community health workers (CHWs) are tasked with following up all confirmed incident malaria cases diagnosed at either a health centre or a CHW health post by testing all household members living within 140 m of the incident malaria case with a malaria RDT. All CHWs are literate and are trained repeatedly on both malaria rapid diagnostics and treatment as well as maintaining quality registries. The 140-m radius for conducting RCD was derived from spatial analyses of a mass screening and treatment study in another area of Southern Province [13, 24]. Individuals testing positive are immediately treated with an effective anti-malarial or if presenting with signs and symptoms of severe malaria referred to the nearest health centre as per national guidelines.

### Community health worker data

CHWs keep detailed registers in simple notebooks recording activities performed. These records are divided into a passive section describing their routine case management activities of diagnosing and treating malaria cases, and an active section describing their case investigations of how many people were tested for malaria and how many people tested positive. CHW paper registers were collected and data from 2012–2013 was transcribed into a custom-built Microsoft Access database.

### Environmental indices

The topographical position index is a measure of an area’s elevation relative to its neighbours, and can be used to identify valleys, plains, ridges, and slopes at varying scales. On a large scale, broad trends are depicted, while at a smaller scales finer nuances in morphometry can be identified [25]. The topographical position index for the study area at scales of 270, 810, 1980, and 4950 m was calculated using Google Earth Engine and a publicly available, hydrology-adjusted, digital elevation model with a resolution of three arc-seconds (30 m) developed by the World Wildlife Fund. From the same digital elevation model the convergence index (CI) and topographic wetness index (TWI) for the study area were generated using the SAGA Convergence Index and SAGA Topographic Wetness Index tools, respectively, available in Quantum GIS version 2.0.1. The convergence index measures an area’s propensity to pool water by comparing the surrounding area’s aspect, i.e., whether the surrounding areas converge (a basin or pit) or diverge (a ridge or cone). The TWI is the ratio of upslope catchment area to an area’s actual slope and estimates how water flows through an area with higher values associated with wetter soils.

The enhanced vegetation index (EVI) derived from the moderate-resolution imaging spectroradiometer (MODIS) provided estimates of vegetation density around rural health posts and serves as an indicator of available adult mosquito habitat [26]. EVI indices at eight-day intervals from Google Earth Engine were lagged four weeks and matched to the date of the incident malaria case. The median EVI over the study period was also calculated.

Using the raster package [27, 28] in R version 3.2.4 [29], mean values for all environmental indices were extracted to health posts buffered by 5 km in order to account for catchment areas. Unfortunately geo-coordinates of actual case investigations were not available. Based upon distribution of continuous indices, lagged EVI was categorized above and below 0.5, median EVI above and below 0.25, topographical wetness index above and below 10.2, and convergence index above and below 0 for analysis. TPI at various scales was categorized as ridge (>1 standard deviation above 0), valley (<−1 standard deviation below zero), upper slope (0.5–1 standard deviation below zero), lower slope (−0.5 to −1 standard deviation below zero) and flat (−0.5 standard deviations below 0–0.5 standard deviations above zero).

### Analyses

A series of regression analyses were conducted to better understand factors associated with detecting secondary positives during reactive case detection. First, the probability of an RDT-confirmed incident malaria case to be investigated was predicted from various case-level parameters including age, travel history and gender as well as the CHW workload and seasonality using a simple logistic regression with CHW included as a random intercept. Second, RDT-positivity during reactions was estimated as a function of incident malaria case demographics, CHW demographics, remotely sensed environmental indicators, and remotely sensed topographical indices. A mixed effects zero-inflated Poisson regression with health post included as a random intercept and the number of people tested during each reaction included as the offset better fit the distribution of RDT-positives found during case investigations than a Poisson or negative binomial distribution. Third, the probability of individuals testing positive during a reaction was analyzed using a mixed effects logistic regression with reaction included as a random intercept. This model allowed for measuring the effect that order of house testing had on the probability of finding an RDT-positive individual after accounting for incident malaria case demographics, environmental measures and topographical indices. Because household geo-coordinates were not collected during reactions the order of house testing serves as proxy for distance from the incident malaria case household, with the first house tested being the residence of the incident malaria case and subsequent houses tested expanding geographically from there. In all regression models a sinusoidal function accounted seasonality. All analyses were conducted in Stata version 13.1; a p value <0.05 was considered statistically significant.

## Results

From 2012–13, 333 CHWs saw a total of 23,716 treatment-seeking patients of which 2469 tested positive for a malaria infection (10.4%). Children aged 5–15 years were most likely to present as an incident malaria case (Table 1). RDT-positivity was highly seasonal, peaking at around 10% in the wet season (December–May) then falling below 5% in the dry season (August–November). Of the 2469 confirmed malaria cases that could have been investigated, CHWs investigated 854 (34.6%). A number of factors (Table 2) were associated with the likelihood of investigating incident malaria cases, most notably a higher monthly patient burden decreasing the likelihood of an investigation.

**Table 1:** Rapid diagnostic test positivity among treatment-seeking individuals presenting to community health workers for passive case detection stratified by age

Age (years)	RDT positivity at CHW (95% CI)
<5	5.8% (4.6–6.9%)
5–15	15.8% (12.8–18.7%)
>15	10.4% (9.1–11.6%)

χ^2^ = 77.6, p < 0.0001; *CI* confidence interval

**Table 2:** Factors associated with community health workers conducting case investigations of incident malaria cases

Factor	Categorization	Unadjusted OR (95% CI)	Adjusted OR (95% CI)
Age	15 years or older	Reference	Reference
	5–14 years	0.620*** (0.503–0.766)	0.704** (0.565–0.876)
	<5 years	0.625** (0.661–0.928)	0.694* (0.520–0.925)
Travel	Index case did not travel	Reference	Reference
	Index case travelled	0.701* (0.496–0.990)	0.669* (0.473–0.948)
Gender	Index case is female	Reference	Reference
	Index case is male	0.563*** (0.464–0.682)	0.625*** (0.513–0.761)
Season	During dry season	Reference	Reference
	During rainy season	0.613*** (0.494–0.762)	0.795 (0.628–1.007)
CHW workload	1–6 patients	Reference	Reference
	7–11 patients	0.935 (0.692–1.264)	1.126 (0.824–1.539)
	12–18 patients	0.597** (0.442–0.805)	0.775 (0.563–1.067)
	19–28 patients	0.509*** (0.377–0.687)	0.659* (0.479–0.907)
	29–110 patients	0.496*** (0.349–0.705)	0.650* (0.448–0.943)

N = 2469 cases, 333 CHWs

*CI* confidence interval, *OR* odds ratio

*p < 0.05 **p < 0.01 ***p < 0.001

**Fig. 3:**
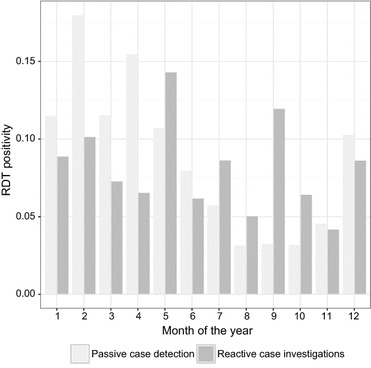
Malaria rapid diagnostic test positivity by month, 2012–2013

The 854 case investigations tested 14,409 individuals during RCD of which 1200 were RDT-positive (8.3%). RDT positivity during reactions exceeded RDT positivity of treatment-seeking individuals during the dry season, but not during the wet season (Fig. 3). A number of factors (Table 3) were associated with increased likelihood of individuals testing positive during case investigations including individual having a fever, living in the index house and being aged 5–15.

**Table 3:** Factors associated with testing positive for malaria during reactive case detection

Factor	Categorization	Unadjusted OR (95% CI)	Adjusted OR (95% CI)
Age	>15 years	Reference	Reference
	5–15 years	1.768*** (1.524–2.053)	1.739*** (1.490–2.031)
	<5 years	1.069 (0.884–1.293)	0.936 (0.768–1.140)
Travel	No travel in previous 2 weeks	Reference	Reference
	Travelled in previous 2 weeks	1.741*** (1.337–2.266)	1.927*** (1.464–2.536)
Gender	Female	Reference	Reference
	Male	1.287*** (1.128–1.469)	1.272** (1.108–1.459)
Season	During dry season	Reference	Reference
	During rainy season	1.403** (1.149–1.714)	1.435** (1.168–1.762)
House location	Beyond nearest neighbours	Reference	Reference
	Index house	3.059*** (2.147–4.359)	2.992*** (2.076–4.312)
	Nearest neighbour (5 houses)	2.065*** (1.465–2.911)	2.033*** (1.426–2.897)
Symptoms	No fever	Reference	Reference
	Fever	4.536*** (3.757–5.475)	4.661*** (3.840–5.657)

N = 14,049 individuals, 859 cases

*CI* confidence interval, *OR* odds ratio

*p < 0.05 **p < 0.01 ***p < 0.001

Just over half of case investigations conducted found no additional malaria infection. All remotely sensed factors were associated with finding positives during case investigations except for the topographical position index at scales of 810 and 1980 m, which were not included in the final analysis because of collinearity with each other (Table 4). Of note, both the convergence index and the topographical wetness index were associated with finding more malaria positive individuals during case investigations. Median EVI over the time-period better predicted the number of positives found than lagged EVI. As a whole, environmental factors rather than incident case demographics better predicted which reactions would find additional malaria infections.

**Table 4:** Factors associated with finding a malaria infection during investigation of an incident malaria case in Southern Province, Zambia

Measure	Categorization	Unadjusted IRR (95% CI)	Adjusted IRR (95% CI)
Topographical position index 270 m	Flat	Reference	Reference
	Valley	1.115 (0.916–1.442)	1.019 (0.733–1.417)
	Ridge	1.268* (1.034–1.554)	1.590* (1.106–2.286)
	Lower slope	0.994 (0.780–1.268)	1.211 (0.893–1.645)
	Upper slope	1.872*** (1.496–2.341)	1.979*** (1.521–2.573)
Topographical position index 4950 m	Flat	Reference	Reference
	Valley	1.000 (0.817–1.224)	1.032 (0.780–1.365)
	Ridge	0.962 (0.782–1.183)	0.576** (0.419–0.792)
	Lower slope	0.732** (0.583–0.919)	0.654** (0.491–0.871)
	Upper slope	1.398** (1.152–1.696)	1.153 (0.909–1.462)
Enhanced vegetation index lagged 4 weeks	<0.5	Reference	Reference
	≥0.5	1.668** (1.209–2.328)	1.291 (0.894–1.865)
Median enhanced vegetation index	<0.25	Reference	Reference
	≥0.25	1.740*** (1.512–2.002)	1.259* (1.049–1.513)
Altitude	≤1100 m	Reference	Reference
	>1100 m	1.442*** (1.252–1.661)	0.949 (0.783–1.151)
Wetness index	≤10.2 (drier)	Reference	Reference
	>10.2 (wetter)	1.061 (0.918–1.225)	1.432*** (1.198–1.712)
Convergence index	≤0 (wetter)	Reference	Reference
	>0 (drier)	1.030 (0.897–1.184)	0.811* (0.665–0.989)
Index case travelled	No travel in previous 2 weeks	Reference	Reference
	Travel in previous 2 weeks	0.898 (0.789–1.188)	0.776 (0.544–1.106)
Timeliness of case investigation	Same week as incident case	Reference	Reference
	At least 1 week following incident case	1.381* (1.072–1.780)	1.211 (0.905–1.621)
Gender of index case	Female	Reference	Reference
	Male	1.329** (1.132–1.560)	0.943 (0.765–1.162)
Travel among individuals in community tested	No travel in previous 2 weeks	Reference	Reference
	Any travel in previous 2 weeks	0.670*** (0.587–0.766)	0.805* (0.668–0.969)
Age of index case	>15 years old	Reference	Reference
	5–15 years old	0.898 (0.740–1.091)	0.862 (0.685–1.085)
	<5 years old	0.761 (0.565–1.025)	0.686* (0.484–0.973)

Zero-inflated Poisson regression models were inflated by the number of people tested and month of the year. Models also controlled for seasonality with a sinusoidal function

N = 690 case investigations

*CI* confidence interval, *IRR* incident rate ratio

*p < 0.05 **p < 0.01 ***p < 0.001

## Discussion

The ability to prioritize intervention responses and target resources where they can have maximum impact will be key to sustaining progress towards elimination and then maintaining this status. In the Zambian context, a key intervention in this quest is community surveillance, including RCD. In this paper, this surveillance system identified 1200 additional malaria infections that did not seek treatment between 2013 and 2014. Despite challenges, these results are encouraging in light of recent articles failing to find utility with RCD, albeit in markedly different settings [8].

Volunteer CHWs investigated approximately one-third of the incident malaria cases, finding additional infections in roughly half of those case investigations. As expected, these volunteers were less likely to perform case investigations during the rainy season and for incident malaria cases reporting travel in the previous two weeks. Furthermore, CHWs investigated fewer malaria cases during months that they had higher workloads in terms of outpatient attendance at the health post. More specific understanding of CHWs in this context is needed to improve CHW productivity in order to pursue malaria elimination with a surveillance system that heavily relies on volunteers [30]. Interestingly, CHWs were less likely to follow up men and children, which may reflect social networks.

Malaria test positivity during case investigations declined as the CHWs moved away from the index case house and sharply fell off after the fifth neighbour tested during the case investigation. Unfortunately, CHWs do not collect geo-coordinates during their routine case investigations and so distance from index cases was not available in the data analyzed. Other research in a nearby area suggests that CHWs may conduct RCD beyond the programmed 140-m radius (Searle et al. in prep.), which would help explain the sharp drop in the malaria positivity. Without geo-coordinates of RCD investigations, geo-coordinates of corresponding health posts were used to calculate the environmental indices. This limitation masks any potential nuances of intra-health post variation in malaria transmission risk. Importantly however, this limitation does not undermine the value of health post location in finding additional malaria infections during RCD. Estimating the mean of the topographical indices would not actually predict mosquito breeding sites as other studies have done [17–20], but rather created a measure of areas around health posts that are more or less likely to harbour mosquito breeding. Further work needs to examine how best to link topography with malaria risk at any given location, perhaps building on mosquito dispersal [31, 32].

To address the lack of geo-coordinates, a word-based georefencing system called ‘What 3 Words’ is being investigated [33]. Briefly, this system applies a grid (3 m × 3 m) across the world’s surface and assigns three words to each grid square. Allowing any location to be identified with just three words, e.g., the entrance to The White House is ‘curve.empty.buzz’. The aim is to distribute the word identifiers to each and every household during campaigns, e.g., indoor residual spraying or net distributions, thereby allowing accurate locations to be recorded when visiting a household for RCD. Depending on the success of this approach, individuals may retain these identifiers for case reporting at the clinic.

Contrary to other findings from Zambia [34], incident malaria cases younger than 5 years were less likely to predict malaria positives during case investigation than children aged 5–15 years and were no different from individuals >15 years old. This discrepancy may be explained by the difference in prevalence, and therefore underlying transmission intensities, of 45% RDT positivity in household contacts sampled in Central Province [34] versus 10% reported here. Furthermore, along with individuals reporting symptoms of fever, children aged 5–15 were most likely to test positive for malaria. This age group is known to be the least likely to use insecticide-treated bed nets [35], and may serve as a sentinel population for malaria elimination.

In this analysis, environmental factors proved to be the most important predictors of finding additional malaria infections during RCD. Indeed, four of the five predictive environmental factors separate time-invariant indices (TPI at 270 m, TPI at 4950 m, TWI, CI), and the fifth (median EVI) did not vary over the study period, suggesting that the pockets of residual malaria transmission in this area may be spatially stable at the level of the health post (Fig. 3). This stability is important as it enables targeting of resources towards conventional vector control interventions, e.g., insecticide-treated bed net distribution/utilization, indoor residual spraying or larviciding [36].

## Conclusion

To achieve elimination, residual pockets of transmission must be identified and interventions focused on them. This paper suggests that for RCD responses, the most important factor in identifying which incident cases should be followed up is their location, i.e., environmental risk factors. As reporting becomes more granular and fine-scale risk is better understood, it may be possible to efficiently focus efforts for maximal impact.

## Abbreviations

CHW: community health worker
EVI: enhanced vegetation index
GIS: geographic information system
MODIS: moderate-resolution imaging spectroradiometer
RCD: reactive case detection
RDT: rapid detection test
TPI: topographical position index
TWI: topographical wetness index
